# Adaptive Orbital Rendezvous Control of Multiple Satellites Based on Pulsar Positioning Strategy

**DOI:** 10.3390/e24050575

**Published:** 2022-04-19

**Authors:** Qiang Chen, Yong Zhao, Lixia Yan

**Affiliations:** School of Automation Science and Electrical Engineering, Beihang University (BUAA), Beijing 100191, China; qiangchen@buaa.edu.cn (Q.C.); zhaoyong1996@buaa.edu.cn (Y.Z.)

**Keywords:** pulsar navigation, nonlinear observer, relative satellite dynamics, adaptive control

## Abstract

This paper addresses the orbital rendezvous control for multiple uncertain satellites. Against the background of a pulsar-based positioning approach, a geometric trick is applied to determine the position of satellites. A discontinuous estimation algorithm using neighboring communications is proposed to estimate the target’s position and velocity in the Earth’s Centered Inertial Frame for achieving distributed rendezvous control. The variables generated by the dynamic estimation are viewed as virtual reference trajectories for each satellite in the group, followed by a novel saturation-like adaptive control law with the assumption that the masses of satellites are unknown and time-varying. The rendezvous errors are proven to be convergent to zero asymptotically. Numerical simulations considering the measurement fluctuations validate the effectiveness of the proposed control law.

## 1. Introduction

In recent years, the cooperative rendezvous of orbital satellites has become a popular research topic among the academic community [1]. Advanced positioning and rendezvous control techniques are building a firm base for the potential applications, such as orbital maintenance, orbital refueling, and orbital assembly [2], via steering multiple orbital satellites to achieve rendezvous at a certain target. Particularly, research on the pulsar-based cooperative rendezvous control is of practical significance as the pulsar sources feature high-precision yet stable timing properties for determining the position coordinates of orbital vehicles [3,4].

The pulsar-based positioning technology allows the orbital vehicles to locate themselves by comparing the received signals from pulsar sources with a database of known pulsars and locations [5]. It is the next-generation navigation technology for orbiting or interplanetary spacecrafts [6] and an alternative calibrated source for GNSS (Global Navigation Satellite System) [7]. Compared with the positioning technique, the control technology plays a more important role as the mission success can only be achieved with a robust control design that provides orbital vehicles with robust properties towards the external disturbance, measurement noise, and modeling uncertainty [8,9,10,11,12,13]. In Ref. [8], the extended Kalman filter is applied to denoising the virtual noisy pulsar signal within Poisson distribution and the state observing criteria of a linearized pulsar model via using pulsar data is also proposed. With the help of orbit information and the long-term observation of a single pulsar, navigation algorithms developed based on the adaptive divided different Kalman filter under scenarios of 1–3 orbital satellites are reported in [9]. For improving the reliability, robust control design features the capacity of overcoming the problem of either parameter variation or external disturbances [10,11,14]. Considering the limitation of fuel storage of orbital spacecraft described by Clohessy–Wiltshire equations of motion, the robust $L_{1}$ control strategy shown in [10] has achieved better fuel efficiency during orbit transferring even with parameter variations. One other fuel optimization strategy based on the saw-like control updating algorithm can be found in [12]. As the command from the ground control station to the orbital satellites features an obvious delay phenomenon, advanced control schemes such as [15] can be applicable to fill this gap. Sometimes the trade-off problem between impulsive efficiency and task complexity should even be predetermined before launching the spacecraft into the space [13]. Though the results reported in [8,9,10,11,12,13,14,15] have solved various control problems, only solutions for a single satellite are developed without considering cooperative control of multiple orbital satellites.

As the complexity increases in space missions [16], the cooperative missions of multiple orbital vehicles have received lots of attention [17,18,19,20,21,22,23]. The formation control of numerous spacecrafts is studied in [17] under the J2 perturbation caused by the oblateness of the Earth. Robotic arms and wheeled mobile robots are generally used for ground algorithm investigations [18,19]. Two ground 6-DOF robotic arms are used for performing lidar-based rendezvous and docking control design for orbital satellites [18], in which the real-time target pose estimation and tracking algorithms are applied. A wheeled satellite simulator testbed with experimental docking discussions is reported in [19], illustrating the basic idea of maneuvering two spacecraft to achieve rendezvous in the final phase. In addition, applying atmosphere lift and drag force for satellites in low Earth orbits is proved to be an alternative to complete rendezvous and docking missions [20]. It is worth noting that the control schemes proposed in [17,18,19,20] convert the orbital formation into a local tracking problem and only achieve the formation control of two satellites. This, however, is not applicable for more general tasks such as orbital assembly that requires multiple orbital vehicles to rendezvous at the target orbit simultaneously [21,22,23]. In Ref. [21], the authors propose an adaptive fuzzy control law that ensures the spacecrafts’ rendezvous errors to be ultimately bounded by small numbers. In Ref. [22], the Lyapunov barrier functions are applied to solve the inner-agent collision avoidance problem of multiple orbital spacecrafts for safe maneuver. Considering the constrained field of view of vision-based devices, the feasible path generating algorithm reported in [23] ensures that the target spacecraft maintains the view of the camera during the rendezvous process. However, the cooperative algorithms developed for multiple satellites in [21,22,23] only achieve small attraction regions of the closed-loop system, i.e., the rendezvous errors must be initialized as tens of meters for maintaining the effectiveness of the control law or the model simplifications. In practice, the orbital vehicles launched for cooperative missions are orbiting in different orbits, which means that the initial distance between them might be hundreds of kilometers, which cannot be dealt with by the control laws reported in [21,22,23]. Additionally, the cooperative control schemes reported in [17,18,19,20,21,22,23] contain the assumption that all orbital vehicles can know the information of the reference target, which is not a necessity for achieving cooperative missions.

Motivated by the discussions above, this paper makes a further endeavor to solve the adaptive rendezvous control problem of multiple uncertain orbital satellites with the background of the pulsar-based positioning method. First, we introduce a direct geometric trick to determine orbital coordinates via assuming simultaneous observations of three pulsars for each satellite. Second, we consider the scenario that only part of the satellites in the group have access to the states of the orbital reference vehicle and propose a discontinuously distributed estimation algorithm to estimate the reference orbital vehicle. Specifically, the estimation errors are globally exponentially convergent to zero. Third, we design a novel saturation-like adaptive control strategy via viewing the estimation algorithm as a virtual reference trajectory for each networked satellite. It is proven in the Lyapunov sense that the rendezvous errors of all networked satellites converge to zero asymptotically.

The main contribution of this paper is presenting the design and analysis of a novel adaptive rendezvous strategy for multiple uncertain orbital satellites with an initial pulsar-based positioning method. In comparison with control schemes developed for the single orbital satellites in [8,9,10,11,12,13] or the rendezvous of two spacecrafts in [17,18,19,20], the cooperative control algorithm in this paper allows numerous satellites to achieve rendezvous at the reference target. The adaptive capacity towards unknown and time-varying masses provided by the presented control scheme also features more practical significance than that only considers constant mass in [23]. Additionally, the proposed rendezvous algorithm still works even if the initial rendezvous errors are initialized in hundreds of kilometers, largely expanding the efficient range of a region of attraction in comparison with the control laws reported in [21,22,23].

The remainder is organized as follows. Section 2 presents some useful preliminaries for control formulation. In particular, the pulsar-based position scheme is introduced. Section 3 elaborates on the designs of the estimation algorithm and adaptive control law. Numerical simulations are included in Section 4. Section 5 concludes the work briefly.

## 2. Preliminaries

### 2.1. Notations and Definitions

In this paper, R denotes the real number set, ∥·∥ is the Euclidean norm, |·| represents the absolute value of a scalar, diag{·} forms a diagonal matrix, $I_{n}$ is an *n*-dimensional identity matrix within a vector, $1_{n}$ is an *n*-dimensional identity row vector, $0_{n}$ denotes an *n*-dimensional zero row vector. For a square matrix, λ(·), $\lambda_{m}\left(\cdot \right)$ and $\lambda_{M}\left(\cdot \right)$ represent the eigenvalue, the smallest and largest eigenvalue, respectively. The time *t* is sometimes omitted without making confusion.

### 2.2. Pulsar-Based Positioning

Position measurement for orbital satellites is essential for the onboard control systems to maintain stability. It highly relies on the Telemetry, Tracking, and Command (TT&C) stations built on the ground or other orbital GNSS satellites, which requires continuous yet enormous investment. In comparison with that, pulsars are natural high-precision timing sources and are suitable for determining the position coordinates of satellites in both orbital and interplanetary missions. Henceforth, using pulsars as positioning sources is of practical significance for rendezvous control.

In what follows, an initial pulsar-based positioning method is presented.

**Assumption** **1.** *The solar system barycenter coincides with the center of mass of the Sun.*

**Assumption** **2.** *The axial precession of the Earth is neglected and the Earth orbit is a pure Keplerian orbit without being affected by other celestial bodies except the Sun.*

**Figure 1:**
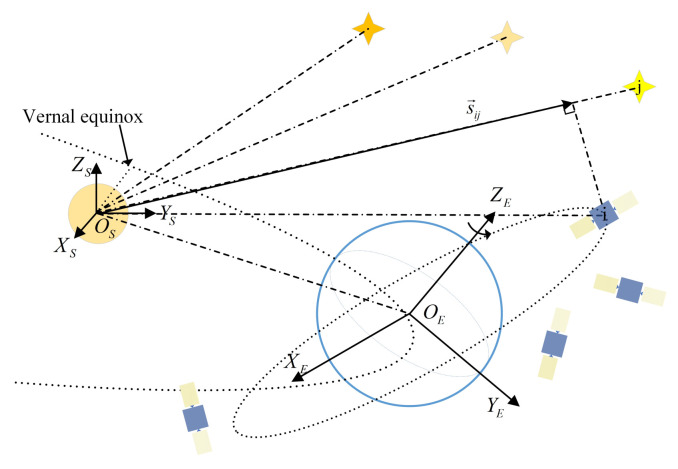
Geometric relationships among pulsars, the Sun, the Earth and the satellites.

With Assumptions 1 and 2, the geometric relationship among pulsars, Sun, Earth, and satellites can be shown in Figure 1. The Sun-Centered-Inertial (SCI) frame $O_{s}-X_{s}Y_{s}Z_{s}$ that Earth is rotating around is viewed as an inertial frame, where $O_{s}$ is the center of mass of the Sun. The $X_{s}$-axis coincides with the direction from the vernal equinox to the $O_{s}$, the $Z_{s}$-axis is perpendicular to the Earth’s orbit plane, and the $Y_{s}$-axis completes the right-hand law according to Z×X. For the Earth-Centered-Inertial (ECI) plane $O_{E}-X_{E}Y_{E}Z_{E}$ that is centered by the Earth, the $X_{E}$-axis features the direction to the vernal equinox, the $Z_{E}$-axis points the rotating axis of the Earth upwards, and the $Y_{E}$ satisfies the right-hand law of $Z_{E}\times X_{E}$. In $O_{s}-X_{s}Y_{s}Z_{s}$ frame, the Earth orbit around the Sun satisfies the following Keplerian dynamics [24],

(1) $${\dot{\vec{r}}}_{s,e}={\vec{v}}_{s,e},{\dot{\vec{v}}}_{s,e}=-\frac{\mu_{s}}{r_{s,e}^{3}}{\vec{r}}_{s,e},$$

where ${\vec{r}}_{s,e}$ and ${\vec{v}}_{s,e}$ denote the position and velocity of the Earth, respectively.

Let $\alpha_{j}$ and $\lambda_{j}$ denote the right ascension and declination in frame $O_{s}-X_{s}Y_{s}Z_{s}$ of the pulsar *j*, respectively. The radiation signal of the pulsar *j* has the following direction [9]:

(2) $${\vec{q}}_{j}=\left[\cos\alpha_{j}\cos\lambda_{j},\cos\alpha_{j}\sin\lambda_{j},\sin\alpha_{j}\right].$$

Suppose that there are *n* satellites and the coordinate of *i*-th satellite is given by row vector ${\vec{r}}_{s,i}=\left[x_{s,i},y_{s,i},z_{s,i}\right]$. Generally, the projection of the ${\vec{r}}_{s,i}$ on the vector ${\vec{q}}_{j}$ is supposed to be measurable [5,24], i.e., ${\vec{s}}_{i,j}=\left({\vec{r}}_{s,i}\cdot {\vec{q}}_{j}\right){\vec{q}}_{j}$ is known, where $r_{s,i}=∥{\vec{r}}_{s,i}∥=c\left(t_{st}-t_{ssb}\right)$ with *c* being the light speed, $t_{st}$ the time instant that the satellite receives the pulsar signal, and $t_{ssb}$ the time of solar system barycenter. Henceforth, the plane perpendicular to the vector ${\vec{s}}_{i,j}$ and passing the *i*-th satellite can be given by:

(3) $${\vec{s}}_{i,j}\cdot \left({\vec{r}}_{s,i}-{\vec{s}}_{i,j}\right)=0.$$

Actually, the position vector of the satellite ${\vec{r}}_{s,i}$ in the $O_{s}-X_{s}Y_{s}Z_{s}$ frame can be uniquely determined via solving the linear equation set in the form of Equation (3) if there are more than three pulsars in the detecting area of the satellite. This is because three non-parallel two-dimensional planes would uniquely determine one single point in the three-dimensional surface [25]. For this sake, we make the following assumption:

**Assumption** **3.** *For each satellite, at least three pulsars’ signals can be observed.*

**Remark** **1.** *Note that the state-of-the-art instruments designed for observing pulsar sources, such as those launched by NICER and XPNAV missions, can only perform observation on a single pulsar during certain time windows. Assumption 3 is built upon future technology wherein the onboard equipment allows the orbital satellites to observe multiple pulsar signals simultaneously, which would be achieved by improving sensor sensitivity and minimization packaging.*

### 2.3. Relative Satellite Dynamics in ECI Frame

Considering *n* orbital satellites under ECI coordinates, let row vectors ${\vec{r}}_{i}=\left[x_{e,i},y_{e,i},z_{e,i}\right]$ and ${\vec{v}}_{e,i}={\dot{\vec{r}}}_{e,i}=\left[{\dot{x}}_{e,i},{\dot{y}}_{e,i},{\dot{z}}_{e,i}\right]$ denote the ECI position and velocity of the *i*-th satellite, respectively. The satellites can generate arbitrary thrust force to control their positions and the position control is decoupled from attitude loop. Via ignoring the J2 perturbation caused by Earth obslateness, the unperturbed orbital satellites can be described by [24]:

(4) $${\dot{\vec{r}}}_{e,i}={\vec{v}}_{e,i},{\dot{\vec{v}}}_{e,i}=-\frac{\mu_{e}}{r_{e,i}^{3}}{\vec{r}}_{e,i}+\frac{\tau_{i}}{m_{i}\left(t\right)},i\in N≜\left\{1,2,\cdots ,n\right\},$$

where $\mu_{e}$ is the gravitational constant of the Earth, $m_{i}\left(t\right)$ denotes the mass of the *i*-the satellite, and $\tau_{i}\in R^{3}$ represents the control input. We suppose that ${\dot{m}}_{i}=\alpha ∥\tau_{i}∥$, where α<0 is a negative constant related to specific impulse coefficient. Via observing Equations (1), (2) and (4), the position of the *i*-th satellite satisfies

(5) $${\vec{r}}_{e,i}={\vec{r}}_{s,i}-{\vec{r}}_{s,e},$$

which reveals the basic principle of orbital satellite positioning by measuring pulsar signals. We assume that the satellites can obtain Earth’s position vector in the SCI frame. In reality, the satellite’s total mass is unknown and decreasing due to generating certain thrust forces and torques via burning the fuel. For this sake, the mass $m_{i}\left(t\right)$ and its ratio of change ${\dot{m}}_{i}\left(t\right)$ are supposed to satisfy:

**Assumption** **4.** *For all i∈N, the mass $m_{i}\left(t\right)$ is an unknown variable and satisfies*

(6) $$m_{i}\left(t\right)\geq {\underline{m}}_{i},$$

*where ${\underline{m}}_{i}$ is a known positive constant, and the ratio of change of mass ${\dot{m}}_{i}\left(t\right)$ is a known non-positive bounded variable and ${\ddot{m}}_{i}\left(t\right)$ is bounded.*

Besides *n* satellites, we suppose that there is one single free-flying reference orbital vehicle indexed by 0 and described by:

(7) $${\dot{\vec{r}}}_{e,0}={\vec{v}}_{e,0},{\dot{\vec{v}}}_{e,0}=-\frac{\mu_{e}}{r_{e,0}^{3}}{\vec{r}}_{e,0}.$$

Actually, the state vector of the satellite described by Equation (7) can be uniquely determined by its orbital elements that include the eccentricity, semimajor axis, inclination, longitude of the ascending node, the argument of perigee, and the true anomaly, as well as other equivalent expressions [24]. For endowing the control formulation with the distributed feature, we assume:

**Assumption** **5.** *There is at least one satellite in N has access to the position and velocity of the 0-th satellite described by Equation (7).*

Construct a local vertical and local horizontal (LVLH) coordinate frame $O_{L}-x_{L}y_{L}z_{L}$ according to position vector ${\vec{r}}_{e,ir}=\left[x_{e,ir},y_{e,ir},z_{i,r}\right]$ and velocity vector ${\vec{v}}_{e,ir}=\left[{\dot{x}}_{e,ir},{\dot{y}}_{e,ir},{\dot{z}}_{i,r}\right]$, where the local $z_{L}$ axis points towards the Earth center, the local $y_{L}$ axis perpendicular downwards to the orbital plane and the local $x_{L}$ axis completes the right hand law from $y_{L}$ to $z_{L}$. Without losing generality, the unit axis vectors can be given as ${\vec{x}}_{L}={\vec{y}}_{L}\times {\vec{z}}_{L},{\vec{y}}_{L}=-\frac{{\vec{r}}_{e,ir}\times {\vec{v}}_{e,ir}}{∥{\vec{r}}_{e,ir}\times {\vec{v}}_{e,ir}∥},{\vec{z}}_{L}=-\frac{{\vec{r}}_{e,ir}}{∥{\vec{r}}_{e,ir}∥}$. The angular velocity of the LVLH frame $O_{L}-x_{L}y_{L}z_{L}$ with respect to the ECI frame is:

(8) $${\vec{\omega }}_{ir}=\frac{{\vec{r}}_{e,ir}\times {\vec{v}}_{e,ir}}{r_{e,ir}^{2}},$$

where $r_{e,ir}=∥{\vec{r}}_{e,ir}∥$. Define the relative position between the *i*-th satellite and ${\vec{r}}_{e,ir}$ in $O_{L}-x_{L}y_{L}z_{L}$ frame by ${\vec{r}}_{ir}=\left({\vec{r}}_{e,i}-{\vec{r}}_{e,ir}\right)\cdot {\left[{\vec{x}}_{L};{\vec{y}}_{L};{\vec{z}}_{L}\right]}^{T}$, whose derivative is [24]:

(9) $${\vec{v}}_{ir}≜{\dot{\vec{r}}}_{ir}={\dot{\vec{r}}}_{e,i}-{\dot{\vec{r}}}_{e,ir}-{\vec{\omega }}_{ir}\times {\vec{r}}_{ir}.$$

Then, the associated relative acceleration can be given by:

(10) $$\begin{array}{cc} {\dot{\vec{v}}}_{ir} & =-\frac{\mu_{e}}{r_{e,i}^{3}}{\vec{r}}_{e,i}+\frac{\tau_{i}}{m_{i}}+\frac{\mu_{e}}{r_{e,ir}^{3}}{\vec{r}}_{e,ir}-{\dot{\vec{\omega }}}_{ir}\times {\vec{r}}_{ir}-{\vec{\omega }}_{ir}\times \left({\vec{\omega }}_{ir}\times {\vec{r}}_{ir}\right)-2{\vec{\omega }}_{ir}\times {\vec{v}}_{ir}, \end{array}$$

where ${\dot{\vec{\omega }}}_{ir}=-2\frac{{\vec{v}}_{e,ir}{\vec{r}}_{e,ir}^{T}}{r_{e,ir}^{2}}{\vec{\omega }}_{ir}$ denotes the angular acceleration.

### 2.4. Graph Theory

A graph G={N,E,A} can be used to describe the interaction among satellites, where E⊆N×N is the edge set and A denotes the adjacency matrix [26]. An edge $\left\{\left(i,j\right):i\neq j\right\}\in E$ denotes that the *j*-th satellite can send information to the *i*-th satellite via wireless devices. The adjacency matrix is defined by $A=\left\{a_{ij}\right\}\in R^{n\times n}$, where $a_{ij}=1$ if $\left(i,j\right)\in E$, otherwise $a_{ij}=0$. Self connection is not allowed, i.e., $a_{ii}=0,\forall i\in N$. For an undirected graph, $a_{ij}=1\Leftrightarrow a_{ji}=1$ holds, denoting that satellite *i* and satellite *j* can exchange information with each other. The path from satellite *i* to satellite *j* denotes an edge sequence $\left\{\left(i,j_{1}\right),\left(j_{2},j_{3}\right),\cdots ,\left(j_{*},j\right)\right\}$. The graph G is called connected if there is a path between any two distinct nodes. The in-degree matrix is given by: $D=\mathrm{diag}\left\{[l_{11},l_{22},\cdots ,l_{nn}]\right\},l_{ii}=\sum_{j=1}^{n}a_{ij},\forall i,j\in N,$ and the Laplacian matrix is: L=D−A. As reported, the matrix L is semi-positive definite and has only one zero eigenvalue and n−1 positive eigenvalues provided that the graph G is undirected and connected [26]. Define $a_{i0}=1$ if the there is a valid information flow from the spacecraft to the *i*-th satellite, otherwise $a_{i,0}=0$. Define:

(11) $$\begin{array}{cc} H & =L+B, \end{array}$$

where $B=\mathrm{diag}\{\left[a_{10},\cdots ,a_{n0}\right]\}$.

**Assumption** **6.** *The graph G is undirected and connected, and $B\neq 0_{n\times n}$.*

**Lemma** **1.** ([26]). *Under Assumption 6, the matrix H is positive definite*.

### 2.5. Problem Formulation

Until now, the control objective can be formally stated as: given Assumptions 1–6, find a control law $\tau_{i},\forall i\in N$ so that the networked satellites described by Equation (4) achieve rendezvous at the orbit trajectory generated by Equation (7), i.e.,

(12) $$\lim_{t\to \infty }{\vec{r}}_{e,i}-{\vec{r}}_{e,0}=0_{3}.$$

## 3. Control Design and Stability Analysis

### 3.1. Estimation Algorithm

Under Assumption 5, the states of the reference orbital vehicle are not available to all satellites, which might obstruct achieving the control objective Equation (12). To overcome this problem, we plan to construct an algorithm to estimate the $\left[{\vec{r}}_{e,0},{\vec{v}}_{e,0}\right]$ via the neighboring communications among satellites in the ECI frame.

Define ${\vec{\eta }}_{e,ir}=\left[x_{e,ir},y_{e,ir},z_{e,ir}\right]$,${\vec{\varphi }}_{e,ir}=\left[{\dot{x}}_{e,ir},{\dot{y}}_{e,ir},{\dot{z}}_{e,ir}\right]$ and ${\vec{\rho }}_{e,ir}=\left[{\ddot{x}}_{e,ir},{\ddot{y}}_{e,ir},{\ddot{z}}_{e,ir}\right]$ as the estimated position, velocity and acceleration of the reference orbital vehicle for the *i*-th satellite. The updating algorithm for ${\vec{\eta }}_{e,ir},{\vec{\varphi }}_{e,ir},{\vec{\rho }}_{e,ir}$ is designed as:

(13) $$\left\{\begin{array}{cc} {\dot{\vec{\eta }}}_{e,ir} & ={\vec{\varphi }}_{e,ir}\\ {\dot{\vec{\varphi }}}_{e,ir} & ={\vec{\rho }}_{e,ir}\\ {\dot{\vec{\rho }}}_{e,ir} & =-g_{1}{\vec{\varphi }}_{e,ir}-g_{2}{\vec{\rho }}_{e,ir}-g_{3}s_{e,ir}-g_{4}\mathrm{sign}s_{e,ir} \end{array}\right.,$$

where $g_{1}>0,g_{2}>0,g_{3}>0,g_{4}>\underset{t\geq 0}{\sup}∥{\vec{r}}_{e,0}^{\left(3\right)}+g_{1}{\ddot{\vec{r}}}_{e,0}+g_{2}{\dot{\vec{r}}}_{e,0}∥$, sign(·) denotes sign function and

(14) $$\begin{array}{cc} s_{e,ir} & =\sum_{j=1}^{n}a_{ij}\left({\vec{\rho }}_{e,ir}-{\vec{\rho }}_{e,jr}\right)+a_{i0}\left({\vec{\rho }}_{e,ir}-{\ddot{\vec{r}}}_{e,0}\right)+g_{1}\sum_{j=1}^{n}a_{ij}\left({\vec{\varphi }}_{e,ir}-{\vec{\varphi }}_{e,jr}\right)+g_{1}a_{i0}\left({\vec{\varphi }}_{e,ir}-{\dot{\vec{r}}}_{e,0}\right)\\ \; & +g_{2}\sum_{j=1}^{n}a_{ij}\left({\vec{\eta }}_{e,ir}-{\vec{\eta }}_{e,jr}\right)+g_{2}a_{i0}\left({\vec{\eta }}_{e,ir}-{\vec{r}}_{e,0}\right). \end{array}$$

**Lemma** **2.** *Given Assumption 6, the gains satisfying $g_{1}>0,g_{2}>0,g_{3}>0,g_{4}$*

*$>\underset{t\geq 0}{\sup}∥{\vec{r}}_{e,0}^{\left(3\right)}+g_{1}{\ddot{\vec{r}}}_{e,0}+g_{2}{\dot{\vec{r}}}_{e,0}∥$ and any initial states, the estimation algorithm given by Equations (13) and (14) ensures that:*

(15) $$\lim_{t\to \infty }{\vec{\eta }}_{e,ir}={\vec{r}}_{e,0},\lim_{t\to \infty }{\vec{\varphi }}_{e,ir}={\dot{\vec{r}}}_{e,0},\lim_{t\to \infty }{\vec{\rho }}_{e,ir}={\ddot{\vec{r}}}_{e,0}.$$

**Proof.**  Define 3n-dimensional stacked row vectors,

(16) $$\begin{array}{cc} \eta_{e,r} & ={\left[{\vec{\eta }}_{e,1r},\cdots ,{\vec{\eta }}_{e,nr}\right]}^{T},\varphi_{e,r}={\left[{\vec{\varphi }}_{e,1r},\cdots ,{\vec{\varphi }}_{e,nr}\right]}^{T},\\ \rho_{e,r} & ={\left[{\vec{\rho }}_{e,1r},\cdots ,{\vec{\rho }}_{e,nr}\right]}^{T},s_{e,r}={\left[s_{e,1r},\cdots ,s_{e,nr}\right]}^{T}. \end{array}$$

It then, by Equations (13) and (14) follows that:

(17) $$\left\{\begin{array}{cc} {\dot{\eta }}_{e,r} & =\varphi_{e,r}\\ {\dot{\varphi }}_{e,r} & =\rho_{e,r}\\ {\dot{\rho }}_{e,r} & =-g_{1}\varphi_{e,r}-g_{2}\rho_{e,r}-g_{3}s_{e,r}-g_{4}P_{e}s_{e,r} \end{array}\right.,$$

where

(18) $$P_{e}=\mathrm{diag}\left\{\mathrm{sign}s_{e,1r},\cdots ,\mathrm{sign}s_{e,nr}\right\}\in R^{3n\times 3n}.$$

Define estimation error

(19) $$\varepsilon_{e,r}={\vec{\eta }}_{e,r}-1_{n}⊗{\vec{r}}_{e,0},$$

where ⊗ denotes the Kronecker product. Within Equation (19), the $s_{e,r}$ in Equation (16) can be arranged as:

(20) $$s_{e,r}=H⊗I_{3}\left({\ddot{\varepsilon }}_{e,r}+g_{2}{\dot{\varepsilon }}_{e,r}+g_{1}\varepsilon_{e,r}\right),$$

with time-derivative along the trajectory of Equation (14) being given by:

(21) $$\begin{array}{cc} {\dot{s}}_{e,r} & =H⊗I_{3}\left({\dddot{\vec{r}}}_{e,r}+g_{2}{\ddot{\varepsilon }}_{e,r}+g_{1}{\dot{\varepsilon }}_{e,r}\right)\\ \; & =H⊗I_{3}\left[-g_{3}s_{e,r}-g_{4}P_{e}s_{e,r}-1_{n}⊗\left({\dddot{\vec{r}}}_{e,0}+g_{2}{\ddot{\vec{r}}}_{e,0}+g_{1}{\dot{\vec{r}}}_{e,0}\right)\right]. \end{array}$$

Given Assumption 6 and Lemma 1, we know that the matrix H is positive definite. Choose a Lyapunov candidate as:

(22) $$V_{1}=0.5s_{e,r}^{T}{\left(H⊗I_{3}\right)}^{-1}s_{e,r},$$

which, by the relationship $\lambda \left(H⊗I_{3}\right)=\lambda \left(H\right)$, leads to:

(23) $$\frac{1}{2\lambda_{M}\left(H\right)}∥s_{e,r}{∥}^{2}\leq V_{1}\leq \frac{1}{2\lambda_{m}\left(H\right)}{∥s_{e,r}∥}^{2}.$$

Then, calculate the time-derivative of $V_{1}$ as:

(24) $$\begin{array}{cc} {\dot{V}}_{1} & =-g_{3}s_{e,r}^{T}s_{e,r}-g_{4}s_{e,r}^{T}P_{e}s_{e,r}-s_{e,r}^{T}1_{n}⊗\left({\dddot{\vec{r}}}_{e,0}+g_{2}{\ddot{\vec{r}}}_{e,0}+g_{1}{\dot{\vec{r}}}_{e,0}\right)\\ \; & =-g_{3}s_{e,r}^{T}s_{e,r}-\sum_{i=1}^{n}\left[g_{4}s_{e,ir}\mathrm{sign}s_{e,ir}^{T}+s_{e,ir}{\left({\dddot{\vec{r}}}_{e,0}+g_{2}{\ddot{\vec{r}}}_{e,0}+g_{1}{\dot{\vec{r}}}_{e,0}\right)}^{T}\right] \end{array}$$

Let $\vartheta \overset{\Delta }{\;=}\underset{t\geq 0}{\sup}\left\{∥{\dddot{\vec{r}}}_{e,0}+g_{2}{\ddot{\vec{r}}}_{e,0}+g_{1}{\dot{\vec{r}}}_{e,0}∥\right\}$. The Equation (24) satisfies:

(25) $$\begin{array}{cc} {\dot{V}}_{1} & \leq -g_{3}{∥s_{e,r}∥}^{2}-\sum_{i=1}^{n}\left|s_{e,ir}\right|\left(g_{4}-\vartheta \right)\\ \; & \leq -g_{3}{∥s_{e,r}∥}^{2}. \end{array}$$

Therefore, $s_{e,r}$ would decay to zero with exponential decaying rate $-0.5g_{3}$ [27]. We then transform Equation (20) into

(26) $${\ddot{\varepsilon }}_{e,r}+g_{2}{\dot{\varepsilon }}_{e,r}+g_{1}\varepsilon_{e,r}={\left(H⊗I_{3}\right)}^{-1}s_{e,r}.$$

The vectors $\varepsilon_{e,r}$,${\dot{\varepsilon }}_{e,r}$ and ${\ddot{\varepsilon }}_{e,r}$ will converge to zero exponentially due to $s_{e,r}$ decays to zero exponentially and the matrix $H⊗I_{3}$ is positive definite [28]. By definition Equation (19), the claims in the lemma follows. This completes the proof. □

Via the information exchange with connected satellites, all satellites achieve the estimate of $\left[{\vec{r}}_{e,0},{\vec{v}}_{e,0}\right]$. One should notice that the generated velocity ${\vec{\varphi }}_{e,ir}$ and acceleration ${\vec{\rho }}_{e,ir}$ are bounded because the associated components of the orbital reference vehicle are bounded, and the exponential convergence to zero of the estimation errors. The generated signals ${\vec{\eta }}_{e,ir},{\vec{\varphi }}_{e,ir}$ and ${\vec{\rho }}_{e,ir}$ stated in Lemma 2 will be viewed as a reference signal for the *i*-th satellite for i∈N. As can be seen, the efficacy of the proposed estimation algorithm relies only on partial access to $\left[{\vec{r}}_{e,0},{\vec{v}}_{e,0}\right]$ and undirected connected communications, which gains a more robust property of the group as a whole when compared with the centralized ones [17,18,19,20,21,22,23] that require all satellites in the group to know the information of the reference target.

### 3.2. Feedback Control

In this subsection, we will take the modeling uncertainty into account and design a control law for each satellite i∈N so as to track the reference signal generated by Equations (13) and (14).

Define tracking errors by:

(27) $$\varpi_{e,i}=\left[{\vec{r}}_{e,i}-{\vec{\eta }}_{e,ir}\right]\cdot {\left[\begin{array}{c} \frac{{\vec{\eta }}_{e,ir}\times {\vec{\varphi }}_{e,ir}}{∥{\vec{\eta }}_{e,ir}\times {\vec{\varphi }}_{e,ir}∥}\times \frac{{\vec{\eta }}_{e,ir}}{∥{\vec{\eta }}_{e,ir}∥}\\ -\frac{{\vec{\eta }}_{e,ir}\times {\vec{\varphi }}_{e,ir}}{∥{\vec{\eta }}_{e,ir}\times {\vec{\varphi }}_{e,ir}∥}\\ -\frac{{\vec{\eta }}_{e,ir}}{∥{\vec{\eta }}_{e,ir}∥} \end{array}\right]}^{T}.$$

Following the derivations Equations (8)–(10), the first- and second-order derivatives of Equation (27) can be calculated as:

(28) $$\begin{array}{cc} {\dot{\varpi }}_{e,i} & ={\dot{\vec{r}}}_{e,i}-\varphi_{e,ir}-\zeta_{e,ir}\times \varpi_{e,i},\\ {\ddot{\varpi }}_{e,i} & =-\frac{\mu_{e}}{r_{e,i}^{3}}{\vec{r}}_{e,i}+\frac{\tau_{i}}{m_{i}\left(t\right)}+\frac{\mu_{e}}{{∥\eta_{e,ir}∥}^{3}}\eta_{e,ir}-{\dot{\zeta }}_{e,ir}\times \varpi_{e,i}-\zeta_{e,ir}\times \left(\zeta_{e,ir}\times \varpi_{e,i}\right)-2\zeta_{e,ir}\times {\dot{\varpi }}_{e,i}, \end{array}$$

where $\zeta_{e,ir}=\frac{\eta_{e,ir}\times \varphi_{e,ir}}{∥\eta_{e,ir}∥}$ and ${\dot{\zeta }}_{e,ir}=-2\frac{\varphi_{e,ir}\eta_{e,ir}^{T}}{r_{e,ir}^{2}}\zeta$ represent the angular velocity and angular acceleration of the frame formed by the reference signal $\left[{\vec{\eta }}_{e,ir},{\vec{\varphi }}_{e,ir}\right]$.

Define an intermediate variable:

(29) $$\begin{array}{cc} \delta_{e,i} & =-\frac{\mu_{e}}{r_{e,i}^{3}}{\vec{r}}_{e,i}+\frac{\mu_{e}}{{∥\eta_{e,ir}∥}^{3}}\eta_{e,ir}-{\dot{\zeta }}_{e,ir}\times \varpi_{e,i}-\zeta_{e,ir}\times \left(\zeta_{e,ir}\times \varpi_{e,i}\right)\\ \; & -2\zeta_{e,ir}\times {\dot{\varpi }}_{e,i}+k_{3}\tanh\left({\dot{\varpi }}_{e,i}+k_{2}\varpi_{e,i}\right)+k_{1}\tanh\left({\dot{\varpi }}_{e,i}\right), \end{array}$$

where $k_{1},k_{2},k_{3}>0$. We then propose the following control law with a parameter updating algorithm,

(30) $$\left\{\begin{array}{cc} \tau_{i} & =-{\hat{m}}_{i}\left(t\right)\delta_{e,i}\\ {\dot{\hat{m}}}_{i} & ={\dot{m}}_{i}\left(t\right)+k_{4}\left[k_{3}\tanh\left({\dot{\varpi }}_{e,i}+k_{2}\varpi_{e,i}\right)+k_{1}\tanh{\dot{\varpi }}_{e,i}+k_{2}{\dot{\varpi }}_{e,i}\right]\delta_{e,i}^{T} \end{array}\right.,$$

where $k_{4}>0$ and ${\hat{m}}_{i}\left(t\right)$ denotes the estimated value for $m_{i}$. The estimated value ${\hat{m}}_{i}\left(t\right)$ is updated via the integration ${\hat{m}}_{i}\left(t\right)={\hat{m}}_{i}\left(0\right)+\int_{0}^{t}{\dot{\hat{m}}}_{i}\left(\chi \right)d\chi$.

**Theorem** **1.** *If the control gains are selected such that $k_{1}>0,k_{2}>0,k_{3}>0,k_{4}>0$, the adaptive control law shown in Equation (30) guarantees that the tracking error $\varpi_{e,i}$ converges to zero globally asymptotically. In addition, the control objective Equation (12) is achieved.*

**Proof.**  Substituting Equation (29) into ${\ddot{\varpi }}_{e,i}$ in Equation (28) results in:

(31) $${\ddot{\varpi }}_{e,i}=-k_{3}\tanh\left({\dot{\varpi }}_{e,i}+k_{2}\varpi_{e,i}\right)-k_{1}\tanh\left({\dot{\varpi }}_{e,i}\right)+\frac{\tau_{i}}{m_{i}\left(t\right)}+\delta_{e,i}.$$

Define estimation error as ${\bar{m}}_{i}\left(t\right)=m_{i}\left(t\right)-{\hat{m}}_{i}\left(t\right)$ and taking the control law Equation (30) into Equation (31), we obtain:

(32) $${\ddot{\varpi }}_{e,i}=-k_{3}\tanh\left({\dot{\varpi }}_{e,i}+k_{2}\varpi_{e,i}\right)-k_{1}\tanh\left({\dot{\varpi }}_{e,i}\right)+\frac{{\bar{m}}_{i}}{m_{i}\left(t\right)}\delta_{e,i}.$$

Choose a positive definite function $V_{2}=V_{2}\left(\varpi_{i},{\dot{\varpi }}_{i},{\bar{m}}_{i},t\right)$ as follows:

(33) $$\begin{array}{cc} V_{2} & =k_{3}m_{i}\ln\;\cosh\left({\dot{\varpi }}_{e,i}+k_{2}\varpi_{e,i}\right)+k_{1}m_{i}\ln\;\cosh{\dot{\varpi }}_{e,i}+0.5k_{2}m_{i}{\dot{\varpi }}_{e,i}{\dot{\varpi }}_{e,i}^{T}+\frac{{\bar{m}}_{i}^{2}}{2k_{4}}. \end{array}$$

The time-derivative of Equation (33) along the solution trajectory of Equation (32) can then be calculated as:

(34) $$\begin{array}{cc} {\dot{V}}_{2} & ={\dot{m}}_{i}\left[k_{3}\ln\;\cosh\left({\dot{\varpi }}_{e,i}+k_{2}\varpi_{e,i}\right)+k_{1}\ln\;\cosh{\dot{\varpi }}_{e,i}+0.5k_{2}{\dot{\varpi }}_{e,i}{\dot{\varpi }}_{e,i}^{T}\right]\\ \; & \;\;\;\;+k_{3}m_{i}\tanh\left({\dot{\varpi }}_{e,i}+k_{2}\varpi_{e,i}\right){\left({\ddot{\varpi }}_{e,i}+k_{2}{\dot{\varpi }}_{e,i}\right)}^{T}+k_{1}m_{i}\tanh{\dot{\varpi }}_{e,i}{\ddot{\varpi }}_{e,i}^{T}+k_{2}m_{i}{\dot{\varpi }}_{e,i}{\ddot{\varpi }}_{e,i}^{T}\\ \; & \;\;\;\;+\frac{{\bar{m}}_{i}}{k_{4}}\left({\dot{m}}_{i}-{\dot{\hat{m}}}_{i}\right). \end{array}$$

As ${\dot{m}}_{i}\leq 0,\forall t\geq 0$, the first row of Equation (34) is non-positive and hence the ${\dot{V}}_{2}$ satisfies:

(35) $$\begin{array}{cc} {\dot{V}}_{2} & \leq k_{3}m_{i}\tanh\left({\dot{\varpi }}_{e,i}+k_{2}\varpi_{e,i}\right){\left({\ddot{\varpi }}_{e,i}+k_{2}{\dot{\varpi }}_{e,i}\right)}^{T}+k_{1}m_{i}\tanh{\dot{\varpi }}_{e,i}{\ddot{\varpi }}_{e,i}^{T}+k_{2}m_{i}{\dot{\varpi }}_{e,i}{\ddot{\varpi }}_{e,i}^{T}\\ \; & \;\;\;\;+\frac{{\bar{m}}_{i}}{k_{4}}\left({\dot{m}}_{i}-{\dot{\hat{m}}}_{i}\right). \end{array}$$

Substitute ${\ddot{\varpi }}_{e,i}$ of Equation (32) into Equation (35),

(36) $$\begin{array}{cc} {\dot{V}}_{2} & \leq m_{i}\left(t\right)\left\{-k_{3}^{2}\tanh\left({\dot{\varpi }}_{e,i}+k_{2}\varpi_{e,i}\right){\left[\tanh\left({\dot{\varpi }}_{e,i}+k_{2}\varpi_{e,i}\right)\right]}^{T}-k_{1}^{2}\tanh{\dot{\varpi }}_{e,i}\tanh{\left({\dot{\varpi }}_{e,i}\right)}^{T}\right\}\\ \; & \;\;\;\;+m_{i}\left(t\right)\left\{-k_{1}k_{2}{\dot{\varpi }}_{e,i}\tanh{\left({\dot{\varpi }}_{e,i}\right)}^{T}-2k_{3}k_{1}\tanh\left({\dot{\varpi }}_{e,i}+k_{2}\varpi_{e,i}\right)\tanh{\left({\dot{\varpi }}_{e,i}\right)}^{T}\right\}\\ \; & \;\;\;\;+\left[k_{3}\tanh\left({\dot{\varpi }}_{e,i}+k_{2}\varpi_{e,i}\right)+k_{1}\tanh{\dot{\varpi }}_{e,i}+k_{2}{\dot{\varpi }}_{e,i}\right]{\bar{m}}_{i}\delta_{e,i}^{T}+\frac{{\bar{m}}_{i}}{k_{4}}\left({\dot{m}}_{i}-{\dot{\hat{m}}}_{i}\right). \end{array}$$

Taking the updating algorithm for ${\hat{m}}_{i}$ in Equation (30) into Equation (36) and performing some direct calculations, we obtain:

(37) $$\begin{array}{cc} {\dot{V}}_{2} & \leq -m_{i}\left(t\right){∥k_{3}\tanh\left({\dot{\varpi }}_{e,i}+k_{2}\varpi_{e,i}\right)+k_{1}\tanh{\dot{\varpi }}_{e,i}∥}^{2}-k_{1}k_{2}m_{i}\left(t\right){\dot{\varpi }}_{e,i}\tanh{\left({\dot{\varpi }}_{e,i}\right)}^{T}\\ \; & \leq -{\underline{m}}_{i}{∥k_{3}\tanh\left({\dot{\varpi }}_{e,i}+k_{2}\varpi_{e,i}\right)+k_{1}\tanh{\dot{\varpi }}_{e,i}∥}^{2}-k_{1}k_{2}{\underline{m}}_{i}{\dot{\varpi }}_{e,i}\tanh{\left({\dot{\varpi }}_{e,i}\right)}^{T}\\ \; & \leq 0. \end{array}$$

It is obvious that ${\dot{V}}_{2}$ is semi-negative definite, which means that $\varpi_{e,i},{\dot{\varpi }}_{e,i}$ and ${\bar{m}}_{i}$ are bounded. With direct calculation, one would find out that $\delta_{e,i}$ is bounded. Henceforth, ${\ddot{\varpi }}_{e,i}$ is bounded. All these bounded variables demonstrate that:

(38) $$\begin{array}{cc} {\ddot{V}}_{2} & ={\ddot{m}}_{i}\left(t\right)\left[k_{3}\ln\;\cosh\left({\dot{\varpi }}_{e,i}+k_{2}\varpi_{e,i}\right)+k_{1}\ln\cosh{\dot{\varpi }}_{e,i}+0.5k_{2}{\dot{\varpi }}_{e,i}{\dot{\varpi }}_{e,i}^{T}\right]\\ \; & \;\;\;\;-{\dot{m}}_{i}\left(t\right){∥k_{3}\tanh\left({\dot{\varpi }}_{e,i}+k_{2}\varpi_{e,i}\right)+k_{1}\tanh{\dot{\varpi }}_{e,i}∥}^{2}-k_{1}k_{2}{\dot{m}}_{i}\left(t\right){\dot{\varpi }}_{e,i}\tanh{\left({\dot{\varpi }}_{e,i}\right)}^{T}\\ \; & \;\;\;\;-2m_{i}\left(t\right)\left[k_{3}\tanh\left({\dot{\varpi }}_{e,i}+k_{2}\varpi_{e,i}\right)+k_{1}\tanh{\dot{\varpi }}_{e,i}\right]\times {\left[\frac{k_{3}{\ddot{\varpi }}_{e,i}+k_{3}k_{2}{\dot{\varpi }}_{e,i}}{1+{∥{\dot{\varpi }}_{e,i}+k_{2}\varpi_{e,i}∥}^{2}}+\frac{k_{1}{\ddot{\varpi }}_{e,i}}{1+{∥{\dot{\varpi }}_{e,i}∥}^{2}}\right]}^{T}\\ \; & \;\;\;\;-k_{1}k_{2}m_{i}\left(t\right){\ddot{\varpi }}_{e,i}\tanh{\left({\dot{\varpi }}_{e,i}\right)}^{T}-k_{1}k_{2}m_{i}\left(t\right)\frac{{\dot{\varpi }}_{e,i}{\ddot{\varpi }}_{e,i}^{T}}{1+{∥{\dot{\varpi }}_{e,i}∥}^{2}} \end{array}$$

is bounded. The boundedness of ${\ddot{V}}_{2}$ implies that ${\dot{V}}_{2}$ is uniformly continuous, which, according to Barbalat’s lemma [27], proves that $\varpi_{e,i}$ and ${\dot{\varpi }}_{e,i}$ converge to zero globally asymptotically. Rewrite Equation (27) as follows:

(39) $${\vec{r}}_{e,i}-{\vec{r}}_{e,0}=\varpi_{e,i}+\left({\vec{\eta }}_{e,ir}-{\vec{r}}_{e,0}\right).$$

Because $\lim_{t\to \infty }\varpi_{e,i}=0$ and $\lim_{t\to \infty }\left({\vec{\eta }}_{e,ir}-{\vec{r}}_{e,0}\right)=0$ hold simultaneously, we use a simple theory of a cascade system [28] and obtain that $\lim_{t\to \infty }\left({\vec{r}}_{e,i}-{\vec{r}}_{e,0}\right)=0$. This completes the proof. □

The above analysis shows that the satellites in N would achieve rendezvous at the reference orbital vehicle. One might note that the safe orbit height is not taken into account, i.e., the satellite must be orbiting above a certain height with respect to the Earth’s surface. We here propose an initial solution for this issue without presenting the detailed proof. Choose two positive numbers $h_{1}$ and $h_{2}$ satisfying $h_{2}>h_{1}>0$, define a continuous and derivable function $f\left(x\right):\left(h_{1},+\infty \right)\mapsto R_{\geq 0}$ as:

(40) $$f\left(x\right)=\left\{\begin{array}{lll} -\alpha_{1}\ln\alpha_{2}\left(x-h_{1}\right)+\alpha_{3} & , & \forall h_{1}<x\leq 0.5\left(h_{1}+h_{2}\right);\\ \alpha_{4}{\left(x-h_{2}\right)}^{2} & , & \forall 0.5\left(h_{1}+h_{2}\right)<x\leq h_{2};\\ 0 & , & \forall h_{2}<x, \end{array}\right.$$

where the constants are chosen as $\alpha_{1}=0.5\alpha_{4}{\left(h_{2}-h_{1}\right)}^{2},\alpha_{2}=\frac{1}{2\left(h_{2}-h_{1}\right)},\alpha_{3}=0.5\alpha_{1},\alpha_{4}>0$. It is direct to verify that f(x) is derivable and continuous for all $x\in (h_{1},+\infty )$, so we omit the proof here. To ensure the orbital satellites orbiting above the $h_{1}$ and $∥{\vec{r}}_{e,i}∥>h_{1},\forall t\geq 0$, we borrow the basic idea from an artificial potential field approach [29] and modify the control law Equation (30) into:

(41) $$\left\{\begin{array}{cc} \tau_{i} & =-{\hat{m}}_{i}\delta_{e,i}+f(∥{\vec{r}}_{e,i}∥)\frac{{\vec{r}}_{e,i}}{∥{\vec{r}}_{e,i}∥}\tau_{i,c}\\ {\dot{\hat{m}}}_{i} & ={\dot{m}}_{i}\left(t\right)+k_{4}\left[k_{3}\tanh\left({\dot{\varpi }}_{e,i}+k_{2}\varpi_{e,i}\right)+k_{1}\tanh{\dot{\varpi }}_{e,i}+k_{2}{\dot{\varpi }}_{e,i}\right]\delta_{e,i}^{T} \end{array}\right.,$$

with $\tau_{i,c}$ being a constant. The introduced term $f(∥{\vec{r}}_{e,i}∥)\frac{{\vec{r}}_{e,i}}{∥{\vec{r}}_{e,i}∥}\tau_{i,c}$ would produce a reversing force in the direction ${\vec{r}}_{e,i}$, pointing outwards from the Earth’s center, so that the $∥{\vec{r}}_{e,i}∥>h_{1},\forall t\geq 0$. In addition, as can be seen from Equations (40) and (41), the term becomes efficient only when $∥{\vec{r}}_{e,i}∥\in \left(h_{1},h_{2}\right)$. This means that the modified control law Equation (41) reduces to Equation (30) if $∥{\vec{r}}_{e,i}∥\geq h_{2}$.

## 4. Numerical Simulation

This section presents the numerical simulation to validate the proposed control design. Two parts are involved. First, we discuss how to propagate the projection vector ${\vec{s}}_{i,j}$ of the satellite position and calculate the satellite position for feedback control. Second, we validate the leader estimation algorithm and feed the disturbed state signals into the control loop to verify the effectiveness of the adaptive control algorithm.

### 4.1. Pulsar-Based Positioning and Simulation Setup

For simulation use, we select three pulsars as positioning sources. The pulsar positions are listed in Table 1 and Table 2. The galactic coordinates shown in Table 1 are transformed into SCI coordinates in Table 2 via the online coordinate calculator provided by https://ned.ipac.caltech.edu/coordinate_calculator, accessed on 11 November 2021.

Within the pulsar position in Table 2, we use Equation (2) to calculate the fixed vectors formed by pulsar radiations in the SCI frame as follows:

(42) $$\begin{array}{cc} {\vec{q}}_{1} & =[0.1027,-0.0023,-0.0226],\\ {\vec{q}}_{2} & =[0.0970,-0.0026,-0.0270],\\ {\vec{q}}_{3} & =[0.3917,0.3564,0.6730]. \end{array}$$

As discussed before, the projection of satellite position vector ${\vec{r}}_{s,i},i\in N$ in the SCI frame on ${\vec{q}}_{j},j=\left\{1,2,3\right\}$ is measurable. Given three pulsars, i.e., j=3, we define the measured row vectors for the *i*-th satellite by ${\vec{s}}_{i,1},{\vec{s}}_{i,2},{\vec{s}}_{i,3}$, which can be propagated by the following equations:

(43) $${\vec{s}}_{i,j}=\left[\left({\vec{r}}_{e,i}+{\vec{r}}_{s,e}\right)\cdot {\vec{q}}_{j}\right]{\vec{q}}_{j},j=\left\{1,2,3\right\},$$

where ${\vec{r}}_{e,i}$ and ${\vec{r}}_{s,e}$ are solved by the associated Keplerian equations of motion. Then we use the two-dimensional plane given in Equation (3) and obtain the following linear equations:

(44) $$\left[\begin{array}{c} {\vec{s}}_{i,1}\\ {\vec{s}}_{i,2}\\ {\vec{s}}_{i,3} \end{array}\right]{\vec{r}}_{s,i}^{T}=[∥{\vec{s}}_{i,1}{∥}^{2},∥{\vec{s}}_{i,2}{∥}^{2},∥{\vec{s}}_{i,3}{{∥}^{2}]}^{T}.$$

Equation (44) suggests that the satellite position in the SCI frame using pulsar measurements can be solved by:

(45) $${\vec{r}}_{s,i}={\left[{\left[\begin{array}{c} {\vec{s}}_{i,1}\\ {\vec{s}}_{i,2}\\ {\vec{s}}_{i,3} \end{array}\right]}^{-1}[∥{\vec{s}}_{i,1}{∥}^{2},∥{\vec{s}}_{i,2}{∥}^{2},∥{\vec{s}}_{i,3}{{∥}^{2}]}^{T}\right]}^{T}.$$

**Figure 2:**
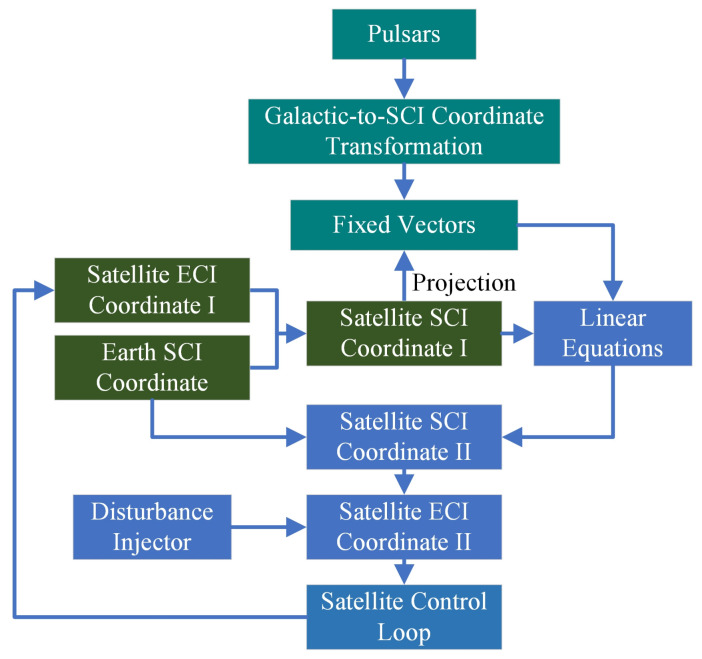
Pulsar-based Positioning Flowchart For Simulations.

To investigate the robust property of the proposed algorithm regarding the disturbance of position and velocity measurement, we inject the unknown time-varying disturbance into the state variables for feedback control. The overall process can be summarized as the figure shown below (Figure 2), in which the satellite ECI coordinate II is fed into the control loop.

### 4.2. Estimate of ${\vec{r}}_{e,0}$ and ${\vec{v}}_{e,0}$

For validating the proposed algorithm, we suppose that four orbital satellites transporting supplies to the international space station (ISS) are indexed by 0, which accords with the rendezvous scenario. The initial orbital elements of ISS and satellites are listed in Table 3.

According to the initial orbital elements in Table 3, we calculate ${\vec{r}}_{e,i}\left(0\right)$ and ${\vec{v}}_{e,i}\left(0\right),\forall i\in N$ as follows:

$$\begin{array}{cc} {\vec{r}}_{e,1}\left(0\right) & =\left[-1880.5,4055.8,5201.9\right]\;\mathrm{km},{\vec{v}}_{e,1}\left(0\right)=\left[-6.3076,-4.1772,1.0317\right]\;\mathrm{km}/s,\\ {\vec{r}}_{e,2}\left(0\right) & =\left[-2082.9,2683.1,6010.5\right]\;\mathrm{km},{\vec{v}}_{e,2}\left(0\right)=\left[-5.9198,-4.7778,0.1278\right]\;\mathrm{km}/s,\\ {\vec{r}}_{e,3}\left(0\right) & =\left[-2669.4,3486.7,5719.6\right]\;\mathrm{km},{\vec{v}}_{e,3}\left(0\right)=\left[-5.6280,-4.8637,0.3940\right]\;\mathrm{km}/s,\\ {\vec{r}}_{e,4}\left(0\right) & =\left[-2493.6,4807.7,4463.1\right]\;\mathrm{km},{\vec{v}}_{e,4}\left(0\right)=\left[-6.2565,-4.1120,1.0216\right]\;\mathrm{km}/s. \end{array}$$

**Figure 3:**
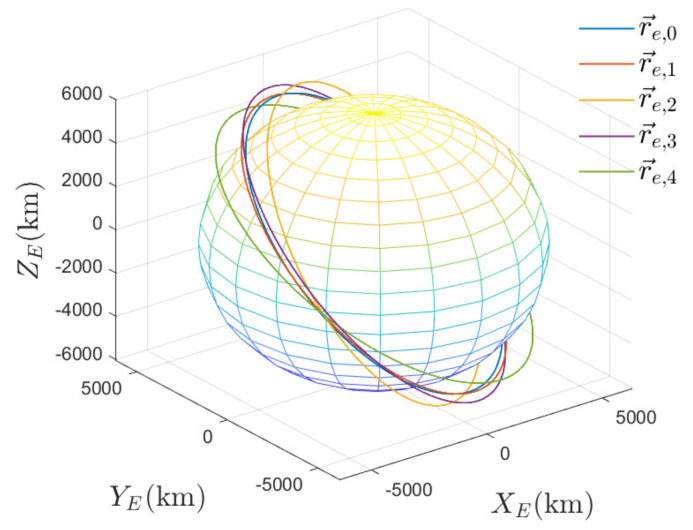
The initial orbits of satellites in the ECI coordinate frame.

The initial orbits of the satellites are depicted in Figure 3. As can be seen, the satellite i,∀i∈N and the ISS are orbiting the Earth with independent orbits.

**Figure 4:**
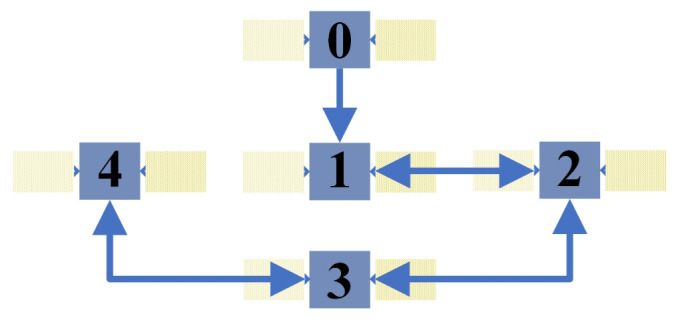
The communication topology.

For achieving distributed estimation about $\left[{\vec{r}}_{e,0},{\vec{v}}_{r,0}\right]$, the satellites in N are supposed to exchange information via the communication topology shown in Figure 4.

**Figure 5:**
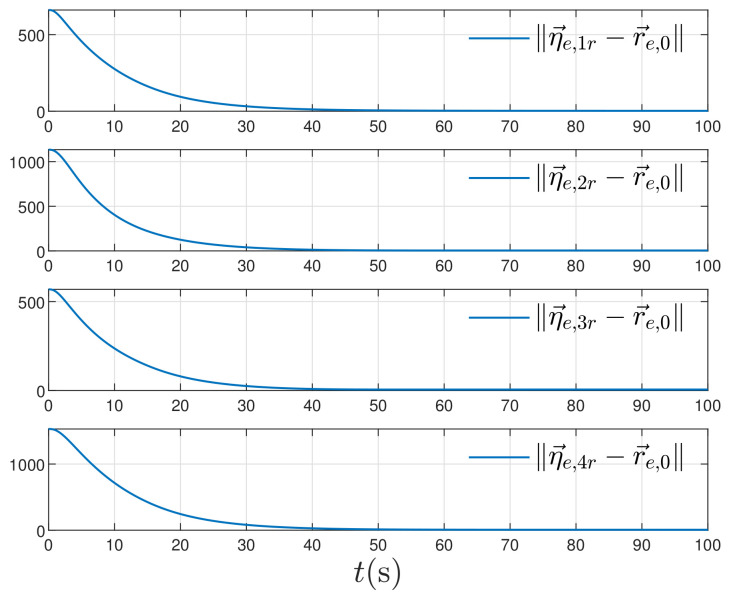
The estimation errors.

The coefficients are chosen as $g_{1}=1,g_{2}=0,1,g_{3}=1,g_{4x}=g_{4y}=g_{4z}=1.2$. Set initial values by ${\vec{\eta }}_{e,ir}\left(0\right)={\vec{r}}_{e,i}\left(0\right),{\vec{\varphi }}_{e,ir}\left(0\right)={\vec{v}}_{e,i}\left(0\right)$ and ${\vec{\rho }}_{e,ir}\left(0\right)=0_{3}$. The simulation time length is the same as the next subsection. However, we only plot the error trajectory in the time interval t∈[0,300] for showing the convergence clearly. The simulation results are depicted in Figure 5. It can be found out that all estimation errors converge to zero asymptotically. Within t≥50 s, the signals ${\vec{\eta }}_{e,1r},\forall i\in N=\left\{1,2,3,4\right\}$ feature stable shape without any chattering phenomenon occurring.

### 4.3. Control Validation

For achieving cooperative tasks, we plan to steer all satellites to the orbit ${\vec{r}}_{e,0}$ via the proposed control law and the pulsar-based positioning method. Within the initial condition shown in Table 3, we set the control coefficients by $k_{1}=0.2,k_{2}=0.4,k_{3}=0.2,k_{4}=0.01$. The real satellite masses are set as $m_{1}=450\;\mathrm{kg},m_{2}=423\;\mathrm{kg},m_{3}=467\;\mathrm{kg}$ and $m_{4}=433\;\mathrm{kg}$. These masses are unknown for each satellite in N. The ratio of change of mass is chosen as $\alpha_{i}=-0.1,\forall i\in \left\{1,2,3,4\right\}$. The initial estimated mass values are set as ${\hat{m}}_{i}\left(0\right)$ = (1 + 6%)$m_{i}$. Set the Sun gravitational constant as $\mu_{s}=1.32712439935\times 10^{11}\;{\mathrm{kg}}^{3}/s^{2}$ and the Earth gravitational constant as $\mu_{e}=3.986005\times 10^{5}\;{\mathrm{kg}}^{3}/s^{2}$. The semi-major axis of Earth rotating round the Sun is chosen as $a_{es}=1.496\;\times \;10^{8}\;\mathrm{km}$ within eccentricity given by $ecc_{e}=0.0167$. The initial anomaly of the Earth is set as zero. The Earth’s radius is set as $R_{e}=6371$ (km). Accordingly, we set $h_{1}=R_{e}+300\;\left(\mathrm{km}\right)=6671\left(\mathrm{km}\right)$, $h_{2}=R_{e}+350\;\left(\mathrm{km}\right)=6721\;\left(\mathrm{km}\right)$, $\alpha_{1}=125,\alpha_{2}=0.04,\alpha_{3}=62.5$, $\alpha_{4}=0.1$ and $\tau_{i,c}=100$. This setting means that we want all satellites are always orbiting 300 km above the Earth’s surface. In addition, the position and velocity measurements used for feedback control are supposed to suffer from disturbances ${\vec{\Delta }}_{e,r,i}$ and ${\vec{\Delta }}_{e,v,i}$ respectively, defined as follows:

$$\begin{array}{cc} {\vec{\Delta }}_{e,r,1} & =50\left[\sin0.01t,\cos0.05t,\sin0.02t\right]\;\left(m\right),{\vec{\Delta }}_{e,r,2}=50\left[\sin0.03t,\sin0.04t,\cos0.02t\right]\;\left(m\right),\\ {\vec{\Delta }}_{e,r,3} & =50\left[\cos0.02t,\sin0.01t,\cos0.03t\right]\;\left(m\right),{\vec{\Delta }}_{e,r,4}=50\left[\sin0.015t,\cos0.02t,\sin0.012t\right]\;\left(m\right),\\ {\vec{\Delta }}_{e,v,1} & =2\left[\sin0.023t,\cos0.03t,\sin0.012t\right]\;\left(m/s\right),{\vec{\Delta }}_{e,v,2}=2\left[\sin0.016t,\sin0.035t,\cos0.022t\right]\;\left(m/s\right),\\ {\vec{\Delta }}_{e,v,3} & =2\left[\cos0.026t,\sin0.013t,\cos0.03t\right]\;\left(m/s\right),{\vec{\Delta }}_{e,v,4}=2\left[\sin0.018t,\cos0.02t,\sin0.032t\right]\;\left(m/s\right). \end{array}$$

**Figure 6:**
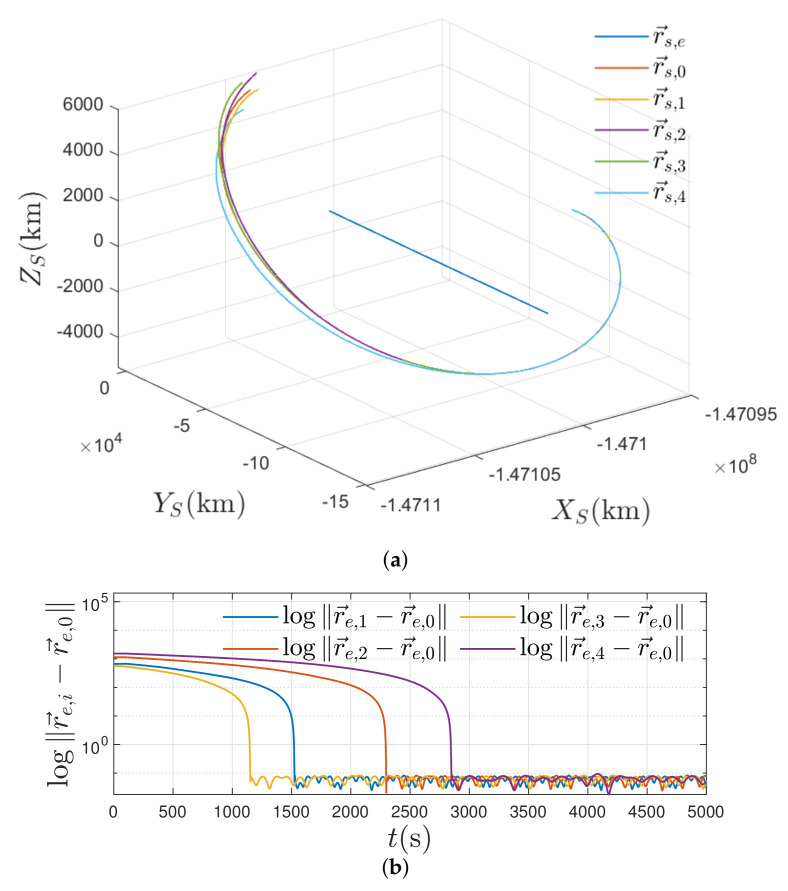
Simulation results of the rendezvous of multiple uncertain satellites. (**a**) The orbits of satellites in the SCI coordinate Frame; (**b**) The position rendezvous errors of semi-logarithmic scale.

**Figure 7:**
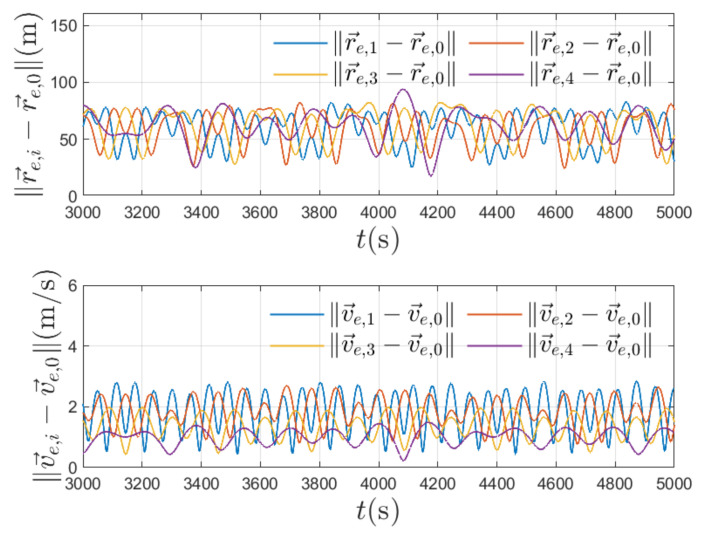
The relative position and velocity in steady state.

**Figure 8:**
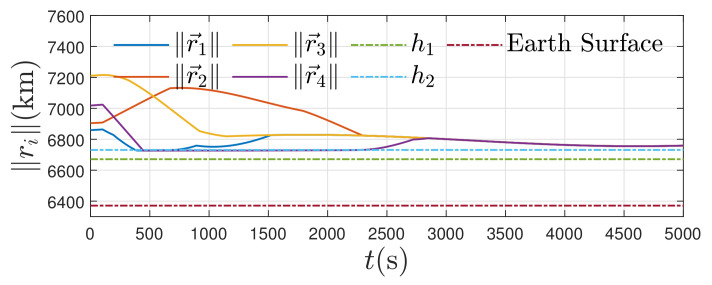
Orbital radius of satellites.

**Figure 9:**
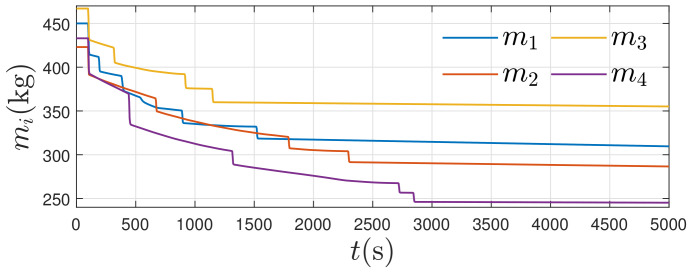
Masses of satellites.

More precisely, the position measurement errors are fluctuated within ±75.7m, while the fluctuation for velocity measurement is ±3.4 m/s. We set simulation time as 5000s and start the control update after t≥100s according to the convergence of the estimation errors stated previously. The simulation results are depicted in Figure 6, Figure 7, Figure 8 and Figure 9.

As depicted in Figure 6a, all satellites in N move to the orbit of ${\vec{r}}_{e,0}$, achieving a rendezvous control scenario from initial orbits. In Figure 6a, the Earth is rotating around the Sun while the satellites are rotating with the Earth. The collective helix curves demonstrate that the control algorithm is effective even though the controller Equation (30) is developed in ECI coordinate frame. Figure 6b further supports this point of view and demonstrates that the position rendezvous errors converge into small regions enclosing the zero.

Due to position and velocity measurement disturbances during the transient phase, the rendezvous errors are not steered converging to zero. We plot the final relative position and velocity with respect to $\left[{\vec{r}}_{e,0},{\vec{v}}_{e,0}\right]$ in Figure 7. The simulation resulting in the steady-state of t≥3000 s shows that the final relative position is maintained to be less than 90 m and the relative velocity to be less than 3 m/s. More precisely, the final relative position and velocity mean values are calculated and summarized in Table 4. As can be seen, the relative position and velocity in the steady-state phase are maintained at a reasonable level. The robust property of the proposed control algorithm towards measurement disturbances is validated. For such small ranges of final relative position and velocity difference, high-precision onboard measurement units can then be used to guide the satellites to accomplish tasks such as docking and circumventing.

By observing Figure 8, we find that the satellites are always over the safe height $h_{1}$, i.e., they would not collide with the Earth’s surface and guarantee $∥{\vec{r}}_{i}∥>h_{1},\forall i\in N,t\geq 0$.

The change of masses due to fuel consumption is plotted in Figure 8. The mass losses are related to the initial rendezvous error. Via simple calculation, we obtain the initial rendezvous error for the first to the fourth satellites as 661.13 km, 1134.40 km, 569.04 km, and 1529.90 km, respectively. These initial errors explain the fuel consumption difference depicted in the figure. Additionally, the masses of satellites for t≥3000 s decrease slightly to overcome the measurement disturbances.

## 5. Conclusions

This paper presents the design and analysis of an adaptive rendezvous control strategy for multiple orbital satellites. Nonlinear tools, including a discontinuous approach, the reduced-order method, and saturation-like adaptive control design, are applied to derive a novel control law that steers multiple uncertain orbital satellites to achieve rendezvous at a certain reference orbital vehicle. It is proven that the rendezvous errors are convergent to zero asymptotically. In the future, the authors will consider more problems for the pulsar-based rendezvous control, such as intermitted observation of pulsar sources, statistical measurements, and fuel optimizations. 

## Figures and Tables

**Table 1:** Pulsar Position in Galactic Frame [30].

*j*	Name	Longitude	Latitude
1	PSR B0531+21	184.56${}^{\circ }$	−5.78${}^{\circ }$
2	PSR B1821−24	7.8${}^{\circ }$	−5.58${}^{\circ }$
3	PSR B1937+21	57.51${}^{\circ }$	−0.29${}^{\circ }$

**Table 2:** Pulsars Information in SCI Frame.

*j*	Name	Right Ascension	Declination
1	PSR B0531+21	84.102438550${}^{\circ }$	−1.29446370${}^{\circ }$
2	PSR B1821−24	275.56845533${}^{\circ }$	−1.54719148${}^{\circ }$
3	PSR B1937+21	301.97479547${}^{\circ }$	42.29726047${}^{\circ }$

**Table 3:** Initial Orbital Elements.

*i*	A	ECC	INCL	RAAN	AANP	APP
0	6792 km	0.005426	51.6438${}^{\circ }$	38.8886${}^{\circ }$	23.0560${}^{\circ }$	63${}^{\circ }$
1	6881 km	0.006340	50.3210${}^{\circ }$	40.0100${}^{\circ }$	20.2022${}^{\circ }$	60${}^{\circ }$
2	6922 km	0.005924	60.5380${}^{\circ }$	39.4500${}^{\circ }$	25.1991${}^{\circ }$	64${}^{\circ }$
3	7238 km	0.007020	52.6225${}^{\circ }$	43.1526${}^{\circ }$	28.5234${}^{\circ }$	58${}^{\circ }$
4	7055 km	0.009070	40.4819${}^{\circ }$	42.5128${}^{\circ }$	23.4040${}^{\circ }$	55${}^{\circ }$

Where the definitions for letters in Table 3 are listed below, A: semi-major axis, ECC: orbit eccentricity, INCL: Inclination, RAAN: right ascension of ascending node, AANP: angle between ascending node and periapsis, APP: angle between periapsis and the satellite.

**Table 4:** The mean values of final relative states for t≥3000 s.

*i*	1	2	3	4
mean$\left\{∥{\vec{r}}_{e,i}-{\vec{r}}_{e,0}∥\right\}$	60.45 m	59.65 m	61.64 m	62.03 m
mean$\left\{∥{\vec{v}}_{e,i}-{\vec{v}}_{e,0}∥\right\}$	1.83 m/s	1.84 m/s	1.36 m/s	1.01 m/s
